# Treatment Interruptions in Chronic HIV Infection

**DOI:** 10.1371/journal.pmed.0010070

**Published:** 2004-12-28

Adverse side effects, viral resistance, and the high cost of antiretroviral therapies remain obstacles in the way of turning HIV/AIDS into a manageable chronic disease. Structured treatment interruptions (STIs) in individuals who have good viral control on therapy have been proposed as a strategy for overcoming these obstacles. The initial hope that STIs would help patients achieve greater viral control has so far not been supported by data from clinical trials, but interrupting treatment has also been proposed as a strategy to reduce the cost of long-term therapy and drug-associated toxicity.

Luis Montaner and colleagues now report results from a randomized trial of 42 participants (75% on their second to fourth regimen, 66% on regimens containing non-nucleoside reverse-transcriptase inhibitors) who received either continuous therapy for 40 weeks or three successive treatment interruptions of two, four, and six weeks, followed by a final open-ended interruption for both groups.

The study was designed to be able to detect a difference of four weeks or greater between the two groups for the time to viral rebound during the open-ended interruption—the primary outcome. No difference between the two groups was seen (median time for the group on continuous treatment was four weeks, and for the STI group was five weeks).

Secondary outcomes included serious adverse events (disease progression, acute retroviral syndrome, therapy failure, or opportunistic infections at any point in the study), changes in CD4 count on therapy, immune reconstitution changes (CD4 recall responses and CD4 naïve/memory T cell distribution), and detection of viral mutations There were no study-related serious adverse events in either group and no increase of therapy failure in the STI arm. CD4 counts fluctuated between the start and end of each monitored treatment interruption, but levels recovered after resuppression of virus, with retention of recall responses throughout. Viral resistance was detected in both groups (in seven of 21 patients in the continuous treatment group and ten of 21 patients in the STI group), but it was more commonly detected (50% versus 18%) in the STI group during the open-ended final interruption, even though all subjects suppressed virus upon reinitiating the same therapy.

Possible risks and benefits of STIs remain controversial, but data from this and other published trials do not support short-term clinical benefits of treatment interruptions. However, because they do not see increased therapy failure and find preservation of immune function in the STI group, the authors conclude that, in light of the possibility of reducing costs and drug-related toxicity, additional trials of STIs are warranted.

Particularly important in the debate over the safety of STIs is whether the detection of resistant mutants should be of concern. The authors point out that all participants were able to resuppress the mutant virus when they resumed their previous drug regimens but state that it remains undetermined to what extent resistant mutations are a signal for future therapy failure. Moreover, viral replication and rebound—which eventually occurred in all participants—is seen by some researchers as inherently detrimental, and these experts argue that treatment interruptions are unsafe and their use should be discontinued.

What seems clear is that STIs have no place outside controlled clinical trials and that questions regarding long-term safety remain unanswered. At least a dozen additional trials that examine STIs are currently recruiting patients and will help answer these questions.

